# Lorcaserin Alters Serotonin and Noradrenaline Tissue Content and Their Interaction With Dopamine in the Rat Brain

**DOI:** 10.3389/fphar.2020.00962

**Published:** 2020-06-30

**Authors:** Giuseppe Di Giovanni, Rahul Bharatiya, Emilie Puginier, Marta Ramos, Salomé De Deurwaerdère, Abdeslam Chagraoui, Philippe De Deurwaerdère

**Affiliations:** ^1^Laboratory of Neurophysiology, Department of Physiology and Biochemistry, Faculty of Medicine and Surgery, University of Malta, Msida, Malta; ^2^School of Biosciences, Neuroscience Division, Cardiff University, Cardiff, United Kingdom; ^3^Centre National de la Recherche Scientifique, UMR CNRS 5287, Bordeaux, France; ^4^Section of Neuroscience and Clinical Pharmacology, Department of Biomedical Sciences, University of Cagliari, Cagliari, Italy; ^5^Normandie Univ, UNIROUEN, INSERM, U1239, CHU Rouen, Neuronal and Neuroendocrine Differentiation and Communication Laboratory, Institute for Research and Innovation in Biomedicine of Normandy (IRIB), Rouen, France; ^6^Department of Medical Biochemistry, Rouen University Hospital, Rouen, France

**Keywords:** monoamines, correlation, brain, high-pressure liquid chromatography, electrochemical detection, cortex, amygdala, nucleus accumbens

## Abstract

Lorcaserin is a preferential serotonin2C receptor (5-HT_2C_R) agonist effective to treat obesity that has also recently been proposed to treat addiction and epilepsy. Central dopamine (DA) mechanisms are likely involved in the lorcaserin mechanism of action, but other monoamines 5-HT and noradrenaline (NA) contents or their interaction with DA might account for its effects. Here we showed that lorcaserin at 3, but not 0.3 mg/kg enhanced 5-HT content in the insular cortex, the core of the nucleus accumbens, and ventral hypothalamus. Without affecting the metabolite 5-hydroxy indole acetic acid, lorcaserin reduced the indirect index of 5-HT turnover in the hippocampus, substantia nigra, and habenula. Lorcaserin at 3 mg/kg increased NA content in the orbitofrontal cortex, the central amygdala (also at 0.3 mg/kg), the ventral hypothalamus, and the shell of the nucleus accumbens. A correlative analysis of the tissue contents between pairs of brain regions revealed that 0.3 mg/kg lorcaserin enhanced the number of correlations for 5-HT, its metabolism, and NA to a lower extent. The correlation profiles were very different between saline, 0.3 and 3 mg/kg lorcaserin. Lorcaserin enhanced the correlations established between NA or 5-HT at 0.3 and 3 mg/kg and reduced the number of correlations established between the index of the turnover for DA and 5-HT. These results show that lorcaserin modulates the biochemistry of NA and 5-HT systems in a subset of brain regions. Qualitatively, they reveal, oppositely to the DA changes, that lorcaserin at 0.3, but not 3 mg/kg, enhanced the number of correlations of 5-HT content between brain regions.

## Introduction

Lorcaserin is a preferential serotonin2C receptor (5-HT_2C_R) agonist that was approved for the treatment of obesity in 2012 (Voigt and Fink, 2015; Greenway et al., 2016; Bohula et al., 2018) but recently withdrawn from clinical use because of long term studies suggesting an increased risk of cancer with its use (https://www.fda.gov/drugs/drug-safety-and-availability/fdarequests-withdrawal-weight-loss-drug-belviq-belviq-xrlorcaserin-market). Several preclinical data have also underscored the possibility that 5-HT_2C_R agonist, including lorcaserin, could reduce the behaviors thought to be mediated by an enhancement of central dopamine (DA) transmission including schizophrenia, and more particularly drug abuse (Higgins et al., 2015; Higgins and Fletcher, 2015; Howell and Cunningham, 2015). The 5-HT_2C_R is an attractive G-protein coupled receptor target in numerous brain diseases, but the mechanisms behind 5-HT_2C_R agonist efficacy are still unclear.

5-HT_2C_Rs are widely expressed in the brain (Hoyer et al., 1986; Abramowski et al., 1995; Eberle-Wang et al., 1997; Clemett et al., 2000). While it was thought that activation of 5-HT_2C_Rs would strongly inhibit the mesencephalic DA neurons (De Deurwaerdere et al., 2004; Higgins and Fletcher, 2015; Howell and Cunningham, 2015; Di Giovanni and De Deurwaerdere, 2016; De Deurwaerdere and Di Giovanni, 2017), some newer electrophysiological and neurochemical evidence has dampened the strength of this control with 5-HT_2C_R agonists lorcaserin or WAY-163909 being less active to inhibit subcortical DA neurons activity (Casarrubea et al., 2015; Pogorelov et al., 2017; Lagiere et al., 2018; De Deurwaerdere et al., 2020a). The data regarding cortical DA release induced by 5-HT_2C_R agonists are even more challenging to reconcile as it has been reported no effect with Ro 60-0175 (Pozzi et al., 2002), decrease with lorcaserin (Pogorelov et al., 2017), and increase with WAY-163909 (Marquis et al., 2007). In keeping in mind that the behavioral results of these newer 5-HT_2C_R agonists parallel those obtained with older agonists except *m*-CPP (Di Giovanni and De Deurwaerdere, 2016), circuits beyond DA neurons should participate in the responses of 5-HT_2C_R agonists.

The serotonergic and noradrenergic systems broadly innervate the brain (Steinbusch, 1984; Aston-Jones, 2004; Hale and Lowry, 2011). 5-HT_2C_R agonists can inhibit the activity of the 5-HT neurons in the dorsal raphe nucleus (DR) (Boothman et al., 2006; Queree et al., 2009), and noradrenaline (NA) neurons in the locus coeruleus (LC) (Gobert et al., 2000; Dekeyne et al., 2008). These findings suggest that 5-HT_2C_R agonists could modulate these two systems either locally or through distal actions in various brain regions and participate in the subtle differences reported between 5-HT_2C_R agonists.

Using postmortem analysis of monoamine tissue contents in a broad number of brain areas, it was found that the preferential 5-HT_2C_R agonist WAY-163909 poorly altered monoamines content quantitatively, but induced changes in the pattern of correlations between pairs of brain regions for monoamines, monoamine metabolism, between monoamines, and between their metabolism (Chagraoui et al., 2019; Whitestone et al., 2019). A similar neurochemical pattern with lorcaserin has been produced recently to determine its action on DA post-mortem index across the brain (De Deurwaerdere et al., 2020a), allowing us to determine whether it alters NA and 5-HT systems alone or in interaction with the DA systems.

In the present study, we report the effects of 0.3 and 3 mg/kg lorcaserin on the quantitative distribution of NA, 5-HT, and its metabolite 5-hydroxyindole acetic acid (5-HIAA) across 30 brain regions of Sprague-Dawley male rats. We furthered the study by performing multiple correlations between pairs of brain regions of the content of NA and 5-HT as well as the index of metabolism 5-HIAA/5-HT. We finally studied the profile of correlations between the neurotransmitters, including DA or between DOPAC/DA and 5-HIAA/5-HT ratios.

## Experimental Procedures

### Animals

Male Sprague Dawley rats weighing 300–400 g were used. They were kept in the animal facility (University of Bordeaux, France) with free access to food and water, in a constant temperature (21 ± 2°C) and humidity (60%) levels, under a 12-h day/night cycle. All the animals’ procedures were performed following the European Council Directive 2010/63/EU and the French National Committee (décret 2001-464) and local committee for the care and use of laboratory animals. All efforts were made to minimize animal suffering and to reduce the number of animals used. In the whole experiment, 30 animals were used, randomly distributed in three experimental groups (n = 10/group).

### Tissue Processing for Post-Mortem Analysis

**Figure 1:**
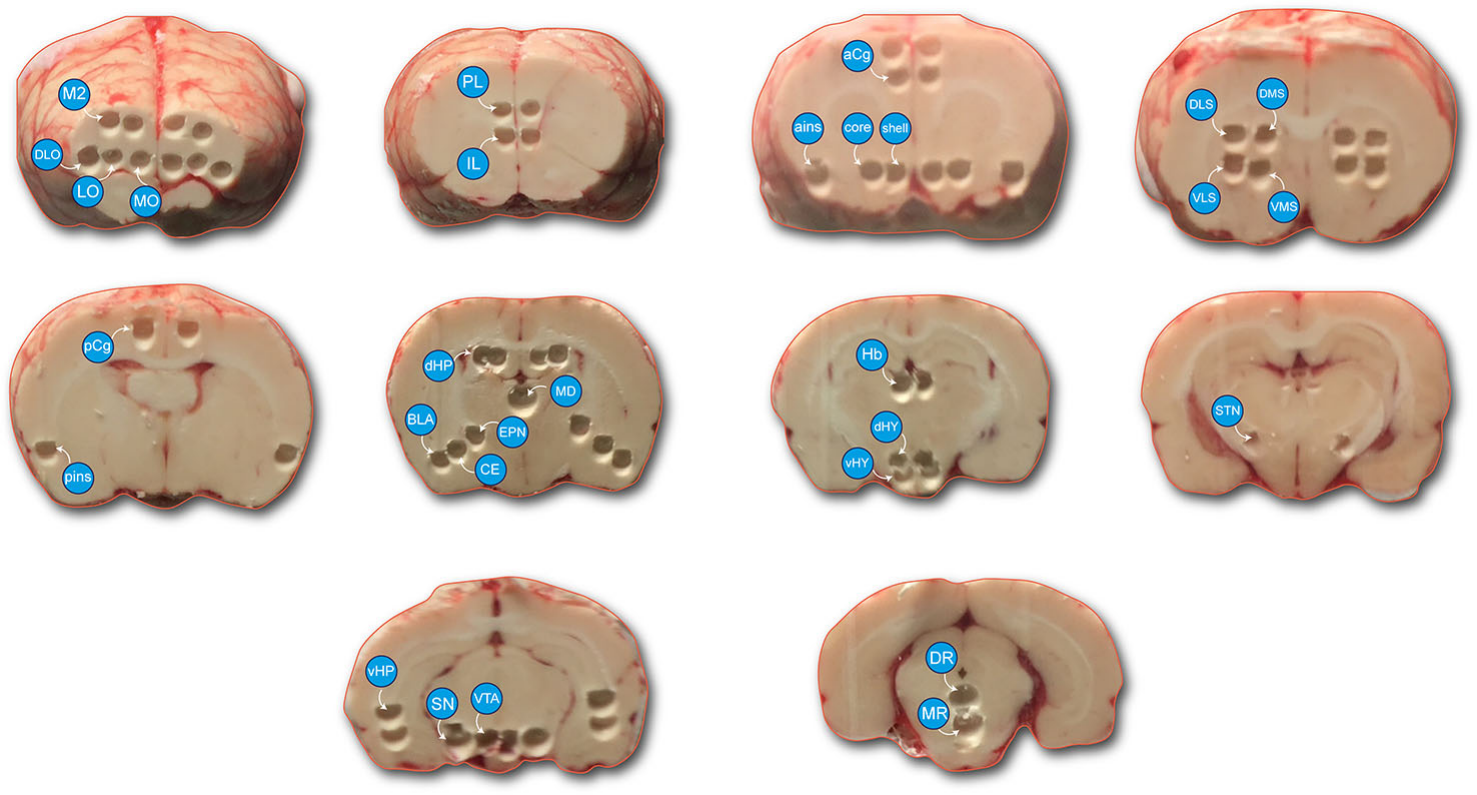
Photomicrograph represents the brain regions sampled. Tissue samples were taken from the left and right cerebral hemispheres in a cryostat. Cortical areas: medial (MO), lateral (LO) and dorsolateral (DLO) orbitofrontal cortex, motor cortex (M2), prelimbic (PL), and infralimbic (IL) cortices, anterior cingulate cortex (aCg), posterior cingulate cortex (pCg), anterior insular cortex (ains), posterior insular cortex (pins), mediodorsal thalamus (MD), dorsal and ventral part of the hippocampus (dHP and vHP), basolateral nucleus (BLA) and central nucleus (CE), nucleus accumbens (shell and core), ventromedial striatum (VMS), ventrolateral striatum (VLS), dorsomedial striatum (DMS), dorsolateral striatum (DLS), entopeduncular nucleus (EPN), subthalamic nucleus (STN), habenula (Hb), dorsal and ventral parts of the hypothalamus (dHY and vHY), substantia nigra (SN), ventral tegmental area (VTA), dorsal raphe nucleus (DR); median raphe nucleus (MR). Two punched tissue in each side were taken from the M2, dHP, vHP regions to be able to measure the concentrations of monoamines (mostly DA and metabolites).

The procedures have been previously published concerning this experiment (De Deurwaerdere et al., 2020a) and are similar to previous ones with some modifications (Fitoussi et al., 2013; Chagraoui et al., 2019). Briefly, the rats were sacrificed 45 min after drug injection (see pharmacological treatment) and the brain was quickly removed. The brains were immersed in isopentane (2-methyl butane) (−35 ± 5°C) and stored in a freezer at −80°C. The brains were cut using a cryostat at −24°C, and bilateral “punches” were made of various brain structures of interest using steel cannulae (**Figure 1**). The STN was taken with a smaller cannula used as a spoon to collect the surface (usually around 200 μm thickness) of the tissue from the medial to the lateral extension of the STN (Chagraoui et al., 2019). **Figure 1** reports actual pictures illustrating the location of the pieces of taken tissues. In some cases, two pieces of tissues from each side (4 at the end) composed the analyzed tissue (M2, dHP, vHP). These punches of tissue were placed in weighed Eppendorf tubes and placed back in the freezer at −80°C. On the day of the biochemical analysis, the tubes containing the samples were weighed again (Dellu-Hagedorn et al., 2017) and 100 μl of perchloric acid (HClO_4_ 0.1N, 4°C) was added. Then, the samples were sonicated for about 6 s and centrifuged at 13,000 rpm for 30 min at 4°C. A volume of 10 or 20 μl (depending on the brain region analyzed) of the supernatant was injected into the high-pressure liquid chromatography (HPLC) system.

### HPLC Analysis and Electrochemical Detection

The tissue concentrations of monoamines were measured by HPLC coupled to the coulometric detection system (Chagraoui et al., 2019). The mobile phase was composed of NaH_2_PO_4_ (70 mM), EDTA (0.1 mM), triethylamine (100 μl/L), sodium octyl sulphate (100 mg/L), and methanol (7%), diluted in deionized water (pH 4.2, adjusted with orthophosphoric acid) as previously reported (De Deurwaerdère et al., 1995). The mobile phase, filtered (0.22 μm) before its installation in the system, was conveyed through the HPLC column (Hypersyl, C18, 15 cm × 4.6 mm, particle size 5 μm, C.I.L.) preceded by a Brownlee-Newgard precolumn (RP-8, 15 mm × 3.2 mm, 7 μm; C.I.L.) using an HPLC pump (LC10Ad Vp, Shimadzu, France) at a 1.2-mL/min flow rate. The aliquots were injected using a manual injection valve (Rheodyne, model 7725i, C.I.L.) equipped with a loop of 20 μl. The elution times of the compounds were approximate as follows (in minutes): NA: 3.30; DOPAC: 4.90; DA: 6.25; 5-HIAA: 9.35; 5-HT: 16.80). The potential of the two electrodes composing the coulometric detection cell (Cell 5011; ESA, Paris, France) was fixed at +350 mV (oxidation) and −270 mV (reduction), respectively, on the electrochemical detector (CoulochemII; ESA, Paris, France). The signals from the detector were recorded on a computer through an interface (Ulyss; Azur System, Toulouse, France).

The calibration curves were adapted according to the brain areas investigated, because the quantities of monoamines and their corresponding metabolites are heterogeneous (Fitoussi et al., 2013), requiring different gains set at the level of the detector using a timeline method. The issue of the sensitivity was marked for DA and its metabolite DOPAC in the hippocampus, the orbitofrontal cortices (even not detectable in this experiment in its dorsolateral part DLO), the hypothalamus (De Deurwaerdere et al., 2020a). NA, 5-HT, and 5-HIAA contents were observed in all sampled regions. The overall sensitivity for the compounds ranged from 2 pg/10 μl for DA to 7 pg/10 μl for 5-HT with a signal/noise ratio of 3:1.

### Pharmacological Treatment and Experimental Design

Lorcaserin is one of the most selective 5-HT_2C_R agonists available in the market, it is brain penetrant and summarizes the main behavioral effects elicited by 5-HT_2C_R agonists in rodents (Thomsen et al., 2008). Lorcaserin (a gift from Dr Andrew J Grottick, Arena Pharmaceuticals, San Diego, United States) was freshly diluted as the salt in NaCl 0.9% and injected i.p. (0.3 or 3 mg/kg). The animals were randomized and received either the drug (0.3 or 3 mg/kg) or vehicle (in 1 ml/kg). Then, quickly we collected the brain areas in order to get the monoamine status at a time corresponding to high behavioral activity (Thomsen et al., 2008; Higgins et al., 2012).

### Statistical Data Analysis

The tissue levels of NA, 5-HT, and 5-HIAA for each of the 30 brain structures were expressed in pg/mg. These levels are presented as the mean ± the standard error of the mean (± SEM) according to their treatment group. The values for DA and DOPAC have been published in a recent article (De Deurwaerdere et al., 2020a).

Outlier data were discarded based on the value outside the range of the average mean ± two standard deviations (Dellu-Hagedorn et al., 2017). Irrespective of the rare outliers, other data were lost for accidental manipulation, loss of chromatographic signals, or aberrant determination of the weight of the sample. The values for NA and 5-HT were compared between experimental groups (saline, lorcaserin at 0.3 or 3 mg/kg, i.p.) with a one-way ANOVA, followed by the Fisher protected least significant difference (PLSD) post-hoc test. A similar analysis was performed for the weight of the tissue between groups for each structure. In all comparisons, p < 0.05 was used as the criterion for significance.

Using the same data of each group for each parameter and brain region, multiple correlative analyses using Bravais-Pearson’s correlation coefficient were performed as qualitative analyses. These analyses were performed within NA and 5-HT systems and between NA, DA, and 5-HT systems in the 30 brain regions (29 when DA was considered because the number of values obtained in the DLO was lost for DA) investigated. As previously reported (Fitoussi et al., 2013), p-values were adjusted using the False Discovering Rate (FDR) controlling procedures (Benjamini and Hochberg, 1995) per group of brain regions. Correlations were then considered as significant at the 5% level and were reported in the corresponding figures.

## Results

### Quantitative Analysis of 5-HT and NA Monoamines After Lorcaserin Treatment

**Figure 2:**
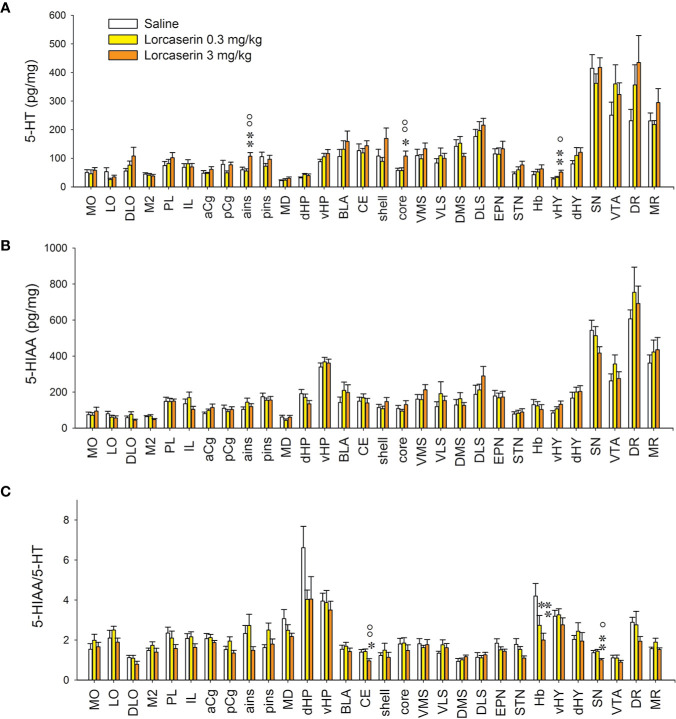
Effect of lorcaserin on 5-HT, 5-HIAA and 5-HIAA/5-HT ratio across the brain. Upper, middle, and lower panels correspond to 5-HT **(A)**, 5-HIAA **(B)** tissue content and 5-HIAA/5-HT ratio **(C)**, respectively. The left, medial, and right bars of histogram correspond to saline-, lorcaserin 0.3 mg/kg, and 3 mg/kg treated rats, respectively. The results correspond to the mean ± SEM of monoamine content (pg/mg tissue) in the 30 different rat brain regions. Lorcaserin has been intraperitoneally administered and the tissue values correspond to 45 min after the administration. The effects of lorcaserin have been compared to saline-treated rats using a one-way ANOVA (see **Table 1** for the number of observations). *p < 0.05, **p < 0.01 with respect to the saline group; °p < 0.05, ^oo^p < 0.01 with respect to the other group of lorcaserin (PLSD’s test).

**Figure 3:**
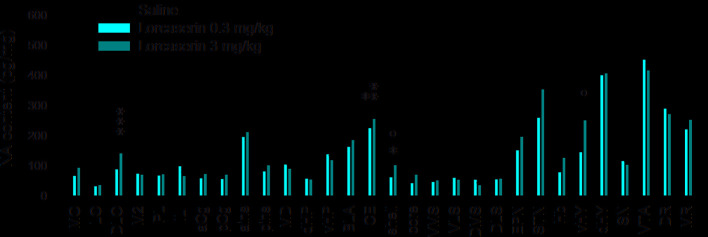
Effect of lorcaserin on NA tissue content across the brain. The left, medial, and right bars of histogram correspond to saline (first column), lorcaserin 0.3 mg/kg (second column), and 3 mg/kg (third column) treated rats, respectively. The results correspond to the mean ± SEM of monoamine content (pg/mg tissue) in the 30 different rat brain regions. Lorcaserin has been intraperitoneally administered and the tissue values correspond to 45 min after the administration. The effects of lorcaserin have been compared to saline-treated rats using a one-way ANOVA (see **Table 1** for the number of observations and ANOVAs). *p < 0.05, **p < 0.01, ***p < 0.001 with respect to the saline group; °p < 0.05 with respect to the other group of Lorcaserin (PLSD’s test).

We have studied the effect of lorcaserin treatment (0.3 and 3 mg/kg, i.p.) on NA and 5-HT system in 30 brain areas of rats and compared it with saline-treated rats. For all brain regions, the size of the tissue did not significantly vary between groups. The quantitative analysis of the effects of lorcaserin for NA and 5-HT is reported in **Figures 2** and **3**.

The tissue levels of 5-HT or its metabolite were very high in mesencephalon regions of the rats (SN, VTA, DR, and MR). Lorcaserin increased the 5-HT levels in the ains, the NAc core, and the vHY only when it was administered at 3 mg/kg (**Figure 2A**, **Table 1**). Lorcaserin did not alter the levels of 5-HIAA, whatever the dose or the brain region analyzed (**Figure 2B**, **Table 1**). Lorcaserin, 3 mg/kg i.p., significantly decreased the 5-HIAA/5-HT ratio in CE and SN as compared to control saline treatment. A substantial decrease was also seen in Hb after lorcaserin injection in a dose-dependent manner (**Figure 2C**, **Table 1**).

**Table 1:** One-way ANOVA results of the effect of lorcaserin on NA, 5-HT, and 5-HIAA tissue content as well as on the ratio 5-HIAA/5-HT.

Brain regions	Abbreviation	Weight (mg ± SEM)	One-way ANOVA			
			NA	5-HT	5-HIAA	5-HIAA/5-HT
***Orbital Cortex***						
medial orbital	**MO**	1.56 ± 0.11	F(2,27)=0.48	F(2,25)=0.28	F(2,25)=0.52	F(2,25)=0.92
Lateral orbital	**LO**	1.55 ± 0.26	F(2,27)=1.08	F(2,26)=2.33	F(2,26)=1.05	F(2,26)=1.21
Dorsolateral orbital	**DLO**	1.43 ± 0.13	F(2,25)=4.57*	F(2,25)=1.91	F(2,24)=2.41	F(2,24)=1.64
Motor M2	**M2**	2.03 ± 0.1	F(2,25)=0.12	F(2,26)=0.38	F(2,26)=2.57	F(2,26)=1.15
***Frontal cortex***						
prelimbic	**PL**	1.08 ± 0.05	F(2,27)=0.31	F(2,27)=0.83	F(2,26)=0.01	F(2,25)=1.86
infralimbic	**IL**	1.4 ± 0.14	F(2,27)=3.02	F(2,27)=0.26	F(2,27)=1.68	F(2,27)=1.62
Anterior cingulate	**aCg**	1.76 ± 0.08	F(2,27)=1.14	F(2,27)=0.93	F(2,27)=1.66	F(2,27)=0.49
Posterior cingulate	**pCg**	1.65 ± 0.09	F(2,26)=1.61	F(2,26)=2.69	F(2,26)=0.40	F(2,26)=3.09
Anterior insular	**ains**	2.06 ± 0.17	F(2,25)=2.43	F(2,23)=7.97**	F(2,22)=1.29	F(2,19)=2.25
Posterior insular	**pins**	1.24 ± 0.08	F(2,27)=0. 70	F(2,27)=1.66	F(2,27)=0.35	F(2,26)=2.87
***Thalamus***	**MD**	1.78 ± 0.09	F(2,27)=0.36	F(2,20)=0.98	F(2,24)=0.87	F(2,19)=2.30
***Hippocampus***						
Dorsal, anterior parts	**dHP**	2.11 ± 0.12	F(2,25)=0.45	F(2,25)=2.12	F(2,24)=1.78	F(2,24)=2.63
Ventral, posterior parts	**vHP**	2.76 ± 0.09	F(2,26)=0.47	F(2,24)=1.52	F(2,26)=0.41	F(2,24)=0.27
***Amygdala***						
Basolateral nucleus	**BLA**	1.13 ± 0.1	F(2,26)=0.71	F(2,26)=0.74	F(2,26)=0.81	F(2,26)=0.46
Central nucleus	**CE**	1.22 ± 0.11	F(2,26)=4.52*	F(2,27)=0.46	F(2,27)=0.40	F(2,25)=4.82*
***Nucleus accumbens-Striatum***						
Shell	**Shell**	1.2 ± 0.08	F(2,25)=3.64*	F(2,27)=2.56	F(2,27)=1.33	F(2,27)=0.52
core	**core**	1.25 ± 0.08	F(2,26)=2.62	F(2,26)=5.35*	F(2,27)=1.09	F(2,26)=0.56
Ventromedial striatum	**VMS**	1.17 ± 0.08	F(2,27)=3.01	F(2,27)=0.89	F(2,27)=1.31	F(2,27)=0.19
Ventrolateral striatum	**VLS**	1.57 ± 0.21	F(2,27)=0.10	F(2,27)=0.40	F(2,27)=0.68	F(2,25)=1.15
Dorsomedial striatum	**DMS**	1.13 ± 0.07	F(2,27)=2.14	F(2,26)=1.49	F(2,27)=0.59	F(2,26)=1.32
Dorsolateral striatum	**DLS**	0.97 ± 0.06	F(2,27)=1.89	F(2,26)=0.54	F(2,26)=1.34	F(2,27)=0.23
***Basal ganglia mesencephalon***						
Entopuncular nucleus	**EPN**	1.64 ± 0.12	F(2,27)=1.02	F(2,22)=0.23	F(2,26)=0.03	F(2,21)=1.58
Subthalamic nucleus	**STN**	1.54 ± 0.08	F(2,26)=1.84	F(2,26)=2.24	F(2,27)=0.10	F(2,26)=3.13
Habenula	**Hb**	1.22 ± 0.07	F(2,27)=2.13	F(2,26)=0.70	F(2,27)=0.28	F(2,26)=5.02*
Substantia nigra	**SN**	1.61 ± 0.13	F(2,24)=0.52	F(2,25)=0.68	F(2,25)=1.81	F(2,25)=5.82**
Ventral tegmental area	**VTA**	1.48 ± 0.07	F(2,24)=0.93	F(2,23)=1.25	F(2,23)=1.31	F(2,23)=0.94
Dorsal raphe nucleus	**DR**	1.53 ± 0.08	F(2,26)=0.68	F(2,26)=1.91	F(2,25)=0.52	F(2,26)=1.33
Median raphe nucleus	**MR**	2.08 ± 0.13	F(2,27)=0.62	F(2,27)=1.49	F(2,27)=0.43	F(2,27)=2.43
***Hypothalamus***						
dorsal parts	**dHY**	1.29 ± 0.08	F(2,27)=0.22	F(2,27)=1.17	F(2,27)=0.44	F(2,27)=0.54
Ventral parts	**vHY**	1.88 ± 0.12	F(2,27)=3.80*	F(2,27)=5.84**	F(2,27)=2.73	F(2,27)=0.76

The table reports the brain structures, their abbreviation, their mean size (in mg), and the ANOVA results of the effect of lorcaserin (0.3 and 3 mg/kg compared with saline treatment) reported on **Figures 2** and **3**. Some data have been lost for different reasons (see methods). *p < 0.05, **p < 0.01.

Similar to 5-HT, the tissue levels of NA varied across the brain of the saline group. Lorcaserin enhanced the NA levels in the DLO and the NAc shell compared to saline-treated rats. Lorcaserin also enhanced NA levels in the CE at both the doses but it did not significantly alter NA tissue levels in other brain regions (one-way ANOVA, ns for all comparisons; **Figure 3**; **Table 1**).

### Qualitative Analysis of Monoamine Tissue Content

#### Within Monoaminergic Systems

**Figure 4:**
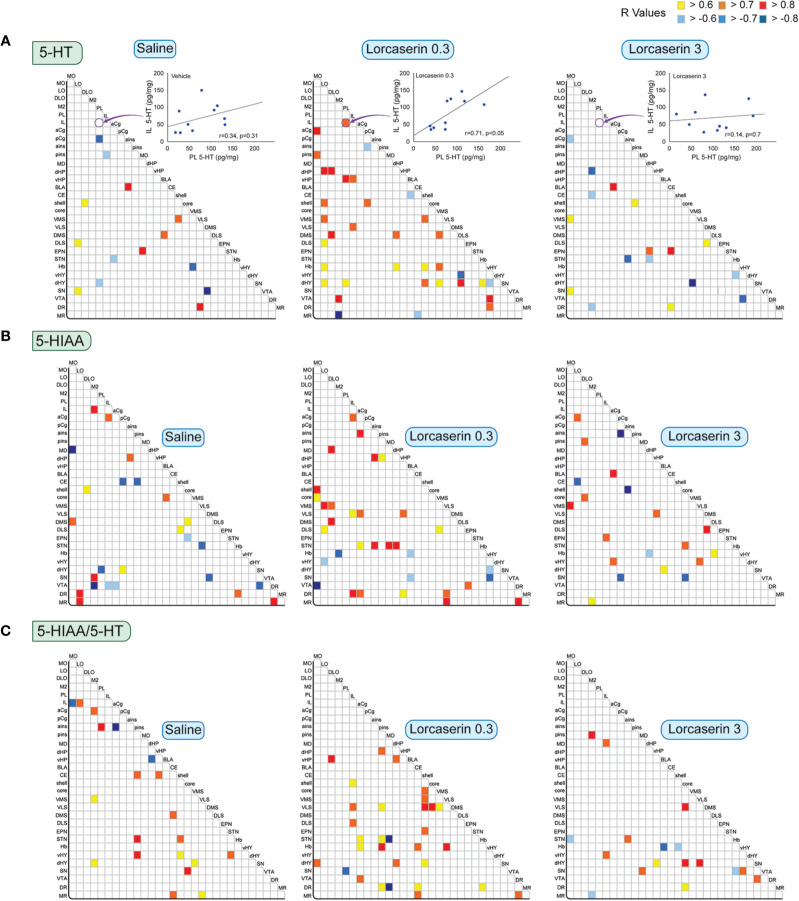
Correlative analysis of 5-HT content across rat brain regions. Representation of the range of Pearson’s R values for each linear regression of 5-HT **(A)**, 5-HIAA **(B)** tissue contents (pg/mg) as well as 5-HIAA/5-HT ratio **(C)** between the 30 brain areas in saline (first column), lorcaserin 0.3 mg/kg (second column), and 3 mg/kg (third column) treated rats. The three insets at the level of 5-HT report one linear regression between the 5-HT content in PL and IL cortices of the animals (n = 10 animals/group) for the three groups. It shows that the 5-HT content in these two brain regions correlates in the lorcaserin 0.3 mg/kg group. These results are reported in the matrix of correlations as indicated by the arrow. Colored boxes correspond to the existence of a correlation between the two parameters (yellow to red, positive; blue, negative) considered after correction for multiple comparisons.

**Figure 4** reports the correlations studied for 5-HT, 5-HIAA, and the ratio 5-HIAA/5-HT between brain regions for the three experimental groups. An example of correlations between 5-HT content in IL and PL is reported at the top. It shows a low, nonsignificant correlation in the saline and lorcaserin 3 mg/kg groups and a higher, significant, and positive correlation in the lorcaserin 0.3 mg/kg group. The saline administration caused only a very few (14) and equal positive (8) and negative correlations (6) between 5-HT content between brain structures (**Figure 4A**). The injection of 0.3 mg/kg lorcaserin increased the number of correlations (36, comprising 6 negative correlations). The 5-HT content in LO and dHY displayed the highest number of correlations with that of other brain regions (6 and 7, respectively). The pattern showed a marked increase in the number of correlations between cortico-subcortical regions and between the Hb or the hypothalamus with subcortical regions. At 3 mg/kg, lorcaserin did not alter much the number of correlations (17, comprising 9 negative correlations) compared to control saline-treated rats but with a different pattern. None of the correlations observed in saline-treated rats were present in lorcaserin 3 mg/kg treated rats.

Likewise, 5-HIAA correlated in 25 brain areas (comprising 14 positive and 11 negative correlations) in saline-treated rats (**Figure 4B**). Interestingly, the number of correlations increased after 0.3 mg/kg lorcaserin administration (33, comprising 27 positive and 6 negative correlations) but decreased after 3 mg/kg lorcaserin (20, comprising 14 positive and 6 negative correlations). The pattern of correlation between lorcaserin treatment groups was drastically different. Lorcaserin at 0.3 mg/kg injection increased the correlations between cortico-striatal regions, including the NAc, but decreased the correlations involving the VTA as compared to control saline-treated rats. The noticeable event was to see the correlations in aCg, STN, and DR (4-5) which were completely absent in control saline-treated rats.

The 5-HIAA/5-HT ratio correlated in 20 brain areas (comprising 17 positive and 3 negative correlations) in saline-treated rats (**Figure 4C**). Lorcaserin at 0.3 mg/kg increased the number of correlations (31 comprising 28 positive and 3 negative correlations), involving NAc/striatal regions. Lorcaserin at 3 mg/kg slightly decreased the number of correlations (17 comprising 5 negative correlations). Only the number of correlations established by the ratio in the SN increased (4).

**Figure 5:**
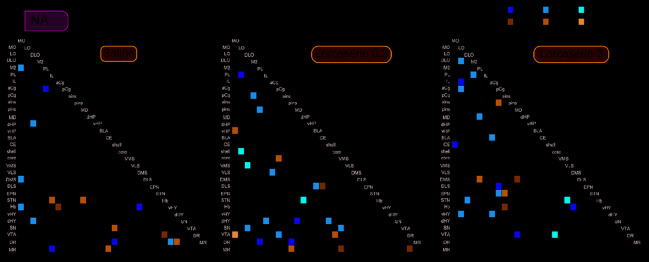
Correlative analysis of NA content across rat brain regions. Representation of the range of Pearson’s R values for each linear regression of NA tissues content (pg/mg) between the 30 brain areas in saline (first column), lorcaserin 0.3 mg/kg (second column), and 3 mg/kg (third column) treated rats. Colored boxes correspond to the existence of a correlation between the two parameters (yellow to red, positive; blue, negative) considered after correction for multiple comparisons.

We observed very few correlations between the NA content of the 30 brain regions in the saline-treated group (17 comprising 10 positive and 7 negative correlations) (**Figure 5**). Lorcaserin at 0.3 and 3 mg/kg increased the number of correlations to 24 and 21, respectively (mostly positive). The NA content established correlation in LO, PL, pCg, dHY, and VTA after lorcaserin treatment (2-3). None of the correlations observed in saline-treated rats were present in lorcaserin-treated rats.

#### Between Monoaminergic Systems

**Figure 6:**
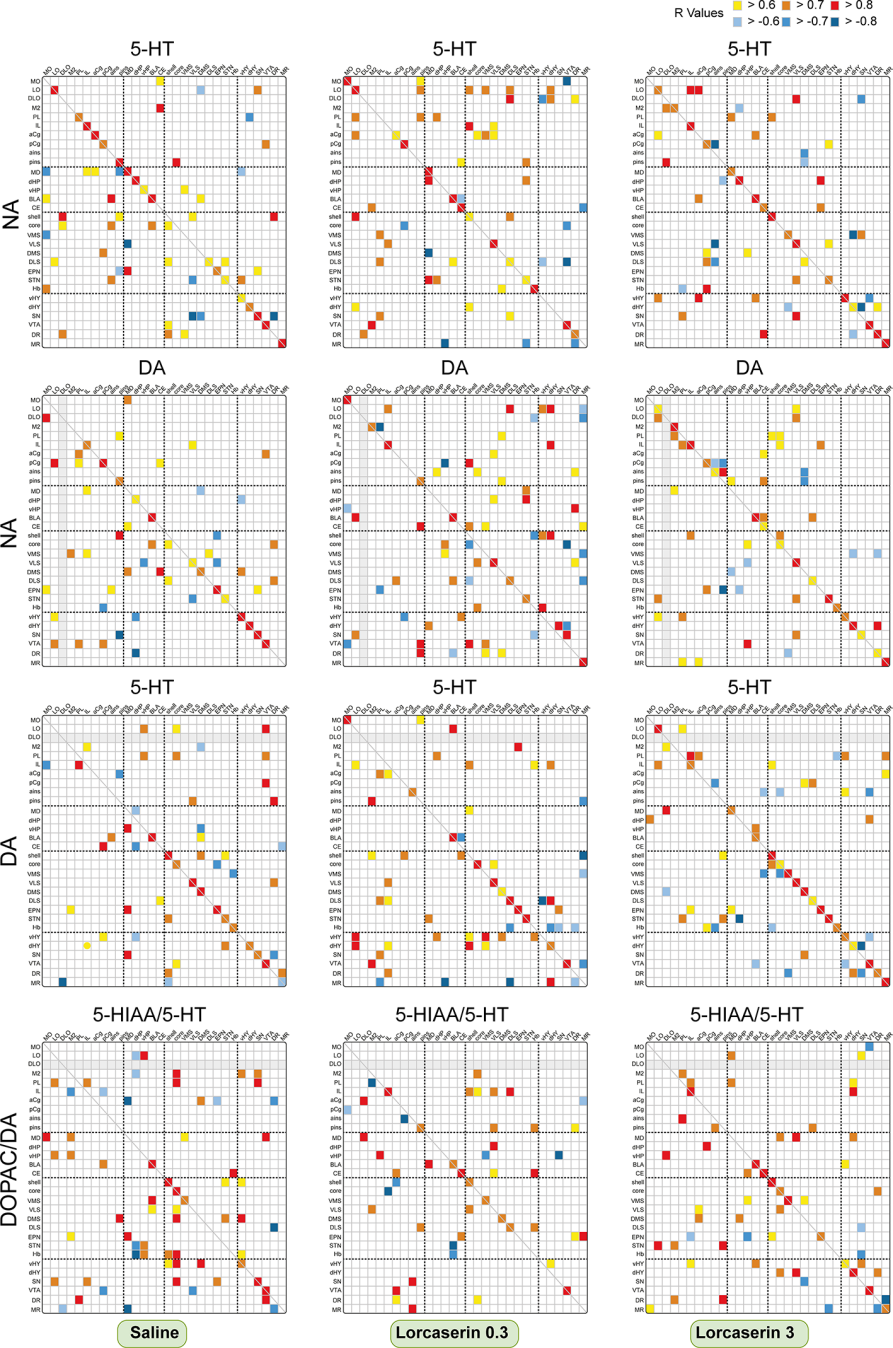
Correlative analysis of monoamine content across rat brain regions. Representation of the range of Pearson’s r values for each linear regression of NA with 5-HT contents (first row), NA with DA contents (second row), DA with 5-HT contents (third row), and the DOPAC/DA and 5-HIAA/5-HT ratios (last row) between the 30 brain areas in saline (first column), lorcaserin 0.3 mg/kg (second column) and 3 mg/kg (third column) treated rats. Colored boxes correspond to the existence of a correlation between the two parameters (yellow to red, positive; blue, negative) considered after correction for multiple comparisons.

We also analyzed possible correlations of the combination of 5-HT with DA, NA with DA and/or the DOPAC/DA with 5-HIAA/5-HT ratios across the brain regions (**Figure 6**). DA and DOPAC/DA values were taken from our recent publication (De Deurwaerdere et al., 2020a).

### Between NA and 5-HT Tissue Content

NA and 5-HT contents diffusively correlated in saline-treated rats with 62 correlations including 18/30 positive ones within the same regions (**Figure 6**). The correlations were mostly found between cortico-striatal and mesencephalon areas of the brain. Lorcaserin did not modify much the number of correlations 65 and 59 (mostly positive) at 0.3 and 3 mg/kg, respectively. At the lowest dose, lorcaserin induced stronger correlations between the NA and 5-HT contents in cortical areas (MO, LO, pins), the NAc (shell and core), the quadrants of striatum except for the DMS. The most numerous correlations between NA content and the 5-HT content were observed in LO (10) with multiple brain regions. Strong positive correlations of NA content from mesencephalon areas with the 5-HT content of orbital cortex (4). We have also seen an increase in the negative correlation between NA content and 5-HT content in the MR. At the highest dose, lorcaserin induced stronger correlation between the NA and 5-HT contents within the orbito-frontal cortices (M2, DLO, IL, aCg, pins, and ains), the NAc (shell and core), the quadrants of the striatum except for the DMS. The hypothalamus regions displayed more correlations in lorcaserin treated rats as compared to saline-treated rats. Lorcaserin also reduced the number of correlations involving the SN.

### Between NA and DA Tissue Content

A total of 53 correlations were observed between NA and DA contents, including 13 in the same brain area (all positive) (**Figure 6**, second row). The number of negative correlations was low (10) compared to saline-treated rats. Lorcaserin at 0.3 mg/kg increased the number of correlations between NA and DA content (69 comprising 52 positive and 17 negative), including 11 in the same brain area. Lorcaserin increased the number of correlations between NA and DA in LO, vHP, NAc shell, Hb, and DR while decreased in pCg. The DA content in the MR negatively correlated with NA content from the orbitofrontal cortices (LO, DLO) and striatum (VMS and DLS). The hypothalamus regions also displayed more correlations compared to saline-treated rats. The pattern of correlation was completely different with lorcaserin 3 mg/kg administration. Lorcaserin treatment slightly changed the number of correlations (57 comprising 46 positive and 11 negative), but increased the number in the same brain area (16). It was noteworthy to observe a complete loss of NA correlation from the VTA with the cortical DA content (LO, PL, and pCg) compared to saline-treated rats. The correlation remained almost unaltered in other brain regions.

### Between DA and 5-HT Tissue Content

The DA and 5-HT correlated in 53 brain areas (comprising 39 positive and 14 negative correlations), including 13 in the same brain area (all positive) in saline-treated rats (**Figure 6**, third row). Interestingly, the number of correlations increased slightly after lorcaserin at both 0.3 and 3 mg/kg injection (61 and 60, respectively, mostly positive correlations). DA content from the hypothalamus and mesencephalon area correlated more with 5-HT content from the orbito-frontal cortices after 0.3 mg/kg lorcaserin. Similarly, we observed a strong positive correlation between the hypothalamic DA content with the 5-HT content in LO, NAc shell, and the striatum compared to saline-treated rats. Additionally, we have seen an increase in negative correlations between DA content and 5-HT content in Hb and MR. There was a significant loss of correlations for the cortical DA content with the 5-HT content in the VTA. The pattern of correlations was completely different for 3 mg/kg lorcaserin. We observed a considerable increase in the number of correlations in orbito-frontal cortices as well as between the frontal cortex and the basal ganglia mesencephalon area.

### Between DOPAC/DA and 5-HIAA/5-HT

The DOPAC/DA and 5-HIAA/5-HT ratio was correlated in 64 brain areas (comprising 49 positive and 15 negative correlations), including 7 in the same brain area (all positive) of saline-treated rats (**Figure 6**, last row). The correlations were distributed in the cortex (LO, M2, PL), hippocampus, NAc (shell and core), Hb, and the mesencephalon area, more in the SN. Interestingly, the number of correlations decreased remarkably after 0.3 and 3 mg/kg lorcaserin injection (48 and 57, respectively, mostly positive correlations). The proportion of correlations in the same brain region was accentuated in lorcaserin treated rats (9 and 11, respectively, all positive). Lorcaserin decreased the correlations of cortical DOPAC/DA ratio with the 5-HIAA/5-HT ratio in the hippocampus (dHP, vHP), the vHY, and the SN. Likewise, there was also a loss of correlations between the DOPAC/DA ratio from the basal ganglia mesencephalon area (EPN, SN, DR, MR) with the 5-HIAA/5-HT ratio in the cortex (LO, DLO, IL, PL). Additionally, there was a complete loss of correlation between the DOPAC/DA ratio from STN, Hb, and vHY with 5-HIAA/5-HT ratio in the hippocampus and the NAc (core and shell). The number of correlations was also reduced in M2, NAc core, STN, Hb, and the SN as compared to saline-treated rats. Lorcaserin 0.3 mg/kg increased the correlations of the DOPAC/DA ratio from infralimbic cortex with the 5-HIAA/5-HT ratio in the NAc and striatum (VLS and DLS). We also observed an increase in the correlations of the DOPAC/DA ratio from the basal ganglia mesencephalon area with the 5-HIAA/5-HT ratio in the frontal cortex (IL and pCg).

## Discussion

In the present study, we report that lorcaserin caused a few quantitative modifications of NA and 5-HT contents across the rat brain, with the main effects being seen in the CE and the vHY for both the neurotransmitter systems. The qualitative study using correlative analyses between pairs of brain regions revealed important reorganization of NA and 5-HT systems after lorcaserin treatment and in the relationship between monoaminergic systems. The study revealed the highest effects in the amygdala, hypothalamus, and the relationships between cortical areas and subcortical regions. Thus, these results further confirm that 5-HT_2C_R activation exerts widespread effects in the brain (Chagraoui et al., 2019; Whitestone et al., 2019; De Deurwaerdere et al., 2020a) and suggests that the effects on NA and 5-HT systems play a role in lorcaserin effects.

Lorcaserin enhanced the NA and 5-HT content in a few brain regions. These effects are unlikely related to the ability of 5-HT_2C_R agonists to alter the activity of NA neurons of the LC or 5-HT neurons of the DRN. Indeed, in both cases, 5-HT_2C_R agonists tend to inhibit the electrical activity of NA (Gobert et al., 2000; Dekeyne et al., 2008) and 5-HT neurons (Boothman et al., 2003; Boothman et al., 2006; Queree et al., 2009). Also, even if an anatomical-functional relationship between NA and 5-HT neurons in the LC and the DRN or MRN has been established (Aston-Jones, 2004; Hale and Lowry, 2011), we report a quantitative modification in a few brain regions which cannot be explained as the sole consequences of a lorcaserin action on the electrical activity of NA and 5-HT neurons. These results confirm that the tissue content of neurotransmitters does not parallel the electrical activity of neurons (De Deurwaerdere et al., 2020a).

Lorcaserin altered the 5-HT metabolic activity in the cortex (here, the anterior insular cortex), the CE of the amygdala, the core, the habenula, and the vHY, similarly to the changes induced on DA brain contents (De Deurwaerdere et al., 2020a). On the same line, lorcaserin increased NA content in the DLO, the CE, the shell, and the vHY. The areas involved in lorcaserin effects are similar for 5-HT, NA, and DA, although the changes do not always reach significance for all the three monoamines. The general profile is different from the one obtained with the preferential 5-HT_2C_R agonist WAY-163909 with the results obtained at the same doses than lorcaserin and corresponding to the same moment after the drug injection. The effects are more tangible with lorcaserin especially for NA for which WAY-163909 did not modify the tissue content in all these brain regions (Chagraoui et al., 2019). Irrespective of the monoamine involved, the CE is the only brain region where neurochemical changes of monoamines have been found with the two agonists, and already at the lowest dose (Chagraoui et al., 2019; Whitestone et al., 2019). This points out that one of the most critical 5-HT_2C_R roles is modulating the amygdala activity (Hackler et al., 2006; Moya et al., 2011; Regue et al., 2019; van de Wouw et al., 2019). It is also not surprising that lorcaserin affects the hypothalamus as the area is involved in the anorexigenic effects of 5-HT_2C_R agonists (Xu et al., 2008; Voigt and Fink, 2015). Finally, the tissue content of all three monoamines is increased either in the shell (NA) or in the core (5-HT and DA) of the NAc, two brain regions which constitute one possible site of drug of abuse (Koob, 1992; Koob et al., 2014) and might account for the suggested anti-addictive properties of lorcaserin (Higgins et al., 2013; Higgins et al., 2015; Higgins and Fletcher, 2015; Howell and Cunningham, 2015).

The effects of lorcaserin on monoamine tissue contents can reach their maximum already at low doses (NA in the CE; 5-HIAA/5-HT in the Hb). The lack of clear dose-response among brain regions is consistent with previous data reported with the preferential though non-selective 5-HT_2C_R agonist m-CPP (Invernizzi et al., 1981). The authors reported a decrease in 5-HIAA that reached significance compared to saline-treated rats 1 h after the injection of 0.3 mg/kg m-CPP. The effects were more sensitive in the brainstem and the telencephalon compared to the striatum or the NAc over the dose-response (0.3–10 mg/kg). Lorcaserin did not modify 5-HIAA content whereas, WAY-163909 either enhanced (CE) or reduced (DLO) 5-HIAA levels only at 0.3 mg/kg (Whitestone et al., 2019). m-CPP acts on several targets other than 5-HT_2C_Rs already at 1 mg/kg (Navailles et al., 2013a) which could account for the lack of clear dose-response as the dose increases. Notwithstanding, its better pharmacological profile toward 5-HT_2C_Rs, the differences between 0.3 and 3 mg/kg lorcaserin could also imply the recruitment of additional sites. Lorcaserin displays a higher affinity for 5-HT_2C_Rs (Ki = 15 nM) compared to 5-HT_2A_Rs (112 nM) and 5-HT_2B_Rs (174 nM, acting as an antagonist) (Thomsen et al., 2008), but previous data have reported that lorcaserin could recruit 5-HT_1A/2A_Rs in behavioral responses such as forepaw treading and drug discrimination (Serafine et al., 2015; Serafine et al., 2016). A brief behavioral evaluation of our rats receiving lorcaserin indicated that rats displayed purposeless oral movements at the low dose, as expected from the 5-HT_2C_Rs agonist profile (Gong et al., 1992; Lagiere et al., 2013; Navailles et al., 2013b; Kreiss and De Deurwaerdere, 2017). However, this effect was lost at the highest dose, and the animals displayed forepaw treading and flat body posture (De Deurwaerdere et al., 2020a). This suggests that higher lorcaserin doses such as 3 mg/kg lose the 5-HT_2C_Rs selectivity (Serafine et al., 2015; Serafine et al., 2016). On the other hand, due to the multiplicity of 5-HT_2C_R subtypes associated with the multiple and complex molecular events [editing, dimerization, coupling to distinct signaling pathways, (Burns et al., 1997; Herrick-Davis et al., 1999; Berg et al., 2001; Chanrion et al., 2008; Herrick-Davis and Farrington, 2011; Herrick-Davis et al., 2015; Berg and Clarke, 2018)] modifying the affinity and efficacy of 5-HT_2C_Rs toward ligands, it is difficult to exclude the recruitment of low efficient 5-HT_2C_Rs as the dose of lorcaserin increases.

The more selective 5-HT_2C_R agonist WAY-163909 (also 0.3 and 3 mg/kg) did not induce clear dose-dependent effects on monoamine contents (Chagraoui et al., 2019; Whitestone et al., 2019). WAY-163909 displays a selective profile toward 5-HT_2C_ receptors among 5-HT_2_R families [Ki = 10.5, 484, and 212 nM at 5-HT_2C_R, 5-HT_2B_R, and 5-HT_2A_R, respectively (Dunlop et al., 2005; Dunlop et al., 2006)], but it can also bind 5-HT_7_R (343 nM) or D_4_R (245 nM) (Dunlop et al., 2005). In addition to the quantitative, regional differences between WAY-163909 and lorcaserin, we found that the number of correlations between monoamines or between the 5-HT/DA metabolisms in a single brain region was lower at 0.3 mg/kg compared to 3 mg/kg lorcaserin. This pattern was opposite with WAY-163909 (Chagraoui et al., 2019) except for the 5-HT/DA metabolism (Whitestone et al., 2019). However, some qualitative effects of WAY-163909 and lorcaserin on the correlations profile of monoamines were similar. Indeed, both compounds reduced the number of correlations for DA to a similar extent at both 0.3 and 3 mg/kg (Chagraoui et al., 2019; De Deurwaerdere et al., 2020a). Moreover, lorcaserin, like WAY-163909, enhanced the number of correlations for NA and 5-HT content at 0.3 and 3 mg/kg. The effects are marked at 0.3 mg/kg for both compounds for the 5-HT system with lorcaserin (this study) and NA system for WAY-163909 (Chagraoui et al., 2019). Thus, while both compounds reduce the number of correlations for DA, they increase the number of correlations on the other monoamines.

The study with the correlations of neurotransmitter content between pairs of brain regions can inform about modified relationships of the content of neurotransmitters at terminals in brain regions (Fitoussi et al., 2013; Dellu-Hagedorn et al., 2017; Puginier et al., 2019; De Deurwaerdere et al., 2020b). Tissue content mostly represents the content of neurotransmitters contained in vesicles of exocytosis while the cytoplasmic content and the extrasynaptic levels would poorly contribute to the total tissue content (Eisenhofer et al., 2004), representing for DA in the striatum 3% and negligible content of total DA, respectively (Pregeljc et al., 2019). Therefore, it is likely that the correlations of monoamine tissue contents reported between brain regions mostly concern the correspondence of their vesicular content. A decrease in the number of correlations for DA tissue content or the ratio DOPAC/DA is not necessarily associated with an increase in the number of correlations for the other systems, as recently reported in the R6/1 mouse model of Huntington’s disease (Puginier et al., 2019). Moreover, an increase in the number of correlations does not imply a stronger influence of the system in the brain, although it suggests that the overall function of NA and 5-HT after 5-HT_2C_R agonists is changing. Recently, we found that low dose haloperidol (10 µg/kg) reduced the number of correlations for the 5-HT system (unpublished data) while this dose of haloperidol recruits plethora of 5-HTRs in the control of striatal DA release (Lucas et al., 2000; Lucas et al., 2001; De Deurwaerdere and Di Giovanni, 2017). A condition can modify the profile of correlations without quantitative changes whereas quantitative changes are expected to modify the correlation profiles (Klouche et al., 2015; Puginier et al., 2019).

It is interesting to develop this approach in situations where the drugs like 5-HT_2C_R agonists are known to modify the function of monoaminergic systems but the corresponding biochemical, extracellular effects using intracerebral microdialysis, are not paralleling the behavioral outcomes (De Deurwaerdere and Di Giovanni, 2017; De Deurwaerdere et al., 2020a). Also, lorcaserin and other 5-HT_2C_Rs agonists reduce motor impulsivity at low doses (Fletcher et al., 2011; Higgins et al., 2012; Higgins et al., 2016; Higgins et al., 2020a), a behavioral property that probably relies on several brain regions involved in cognition (Dalley et al., 2011; Dellu-Hagedorn et al., 2018) including the prefrontal cortex (Anastasio et al., 2014; Anastasio et al., 2015), the orbitofrontal cortex (Alsio et al., 2015), and the NAc (Robinson et al., 2008). The network is larger but depends on the nature of the impulsivity in the impulsive/cognitive dimension and it could involve the CE as well (Dellu-Hagedorn et al., 2018). Here, in terms of correlations at 0.3 mg/kg lorcaserin, we found a higher number of correlations between 5-HT and NA engaging LO, and a higher number of correlations between 5-HT en DA involving PL/IL compared to saline-treated rats, like WAY-163909 at the same dose (Chagraoui et al., 2019). It is noteworthy that the therapeutic dose of lorcaserin (10 mg twice daily) in humans would correspond to 0.3 to 1 mg/kg in rats according to the measured concentrations of lorcaserin in cerebrospinal fluid and plasma of humans and rats (Fletcher et al., 2019; Higgins et al., 2020b).

Lorcaserin was the only preferential 5-HT_2C_R agonist used in therapy (BELVIQ) for its anti-obesity properties (FDA, 2012) but FDA requested on February 13, 2020, its withdrawal from the market for potential risk of cancer outweighs the benefits” (https://www.fda.gov/drugs/drug-safety-and-availability/fda-requests-withdrawal-weight-loss-drug-belviq-belviq-xr-lorcaserin-market). Lorcaserin is also been suggested as an anti-addictive treatment toward drug abuse such as cocaine use disorder and smoking cessation (Arena Pharmaceuticals, 2014; Higgins and Fletcher, 2015; Browne et al., 2017; National Institute on Drug Abuse, 2017). Moreover, some preclinical (Orban et al., 2014; Venzi et al., 2016; Di Maio et al., 2019) and clinical evidence (Tolete et al., 2018) indicates that lorcaserin might be useful for the treatment of different types of epilepsy (see (Di Giovanni and De Deurwaerdere, 2016)). Nevertheless, lorcaserin seems to be effective at high doses when the compound is likely acting largely on sites other than 5-HT_2C_ receptors at 3 mg/kg. Indeed, in the absence epilepsy animal model GAERS, CP-809,101 (3–10 mg/kg) and lorcaserin (3–10 mg/kg) were effective in stopping seizures, an effect only partially blocked by SB242084 (Venzi et al., 2016), a selective 5-HT_2C_ receptor antagonist (Di Matteo et al., 2000). Moreover, lorcaserin 3 mg/kg blocked the elongation of the maximal dentate activation that is an afterdischarge induced by repetitive stimulation of the performant path in the hippocampus, and this effect was not blocked but potentiated by SB242084 (Orban et al., 2014).

While its mechanism remains still unclear, our data suggest that NA and 5-HT systems can participate in its mechanism of action. The changes of monoamine tissue contents for NA and 5-HT share some similarities with those reported for DA and correspond to brain regions associated with drug abuse, obesity, or impulsivity. (Fletcher et al., 2019; Higgins et al., 2020b)Our data suggest that further research on NA and 5-HT involvement in lorcaserin effects could lead to a better understanding of its mechanism of action, and improvement of its efficacy.

## Data Availability Statement

The raw data supporting the conclusions of this article will be made available by the authors, without undue reservation.

## Ethics Statement

The animal study was reviewed and approved by European Council Directive 2010/63/EU and the French National Committee (décret 2001-464) and local committee for the care and use of laboratory animals.

## Author Contributions

PD and GG have conceived the study, designed the methodology, performed data interpretation and supervised the entire process. RB, MR, SD, PD, and EP conducted all the laboratory-based research and performed data interpretation. RB and PD wrote the manuscript and RB, PD, and AC organized the data into tables and figures. GG, PD, and AC have reviewed and edited the manuscript. All authors contributed to the article and approved the submitted version.

## Conflict of Interest

The authors declare that the research was conducted in the absence of any commercial or financial relationships that could be construed as a potential conflict of interest.

## Abbreviations

aCg, anterior cingulate cortex; ains, anterior insular cortex; BLA, basolateral nucleus of the amygdala; CE, central nucleus of the amygdala; core, core of the nucleus accumbens; DA, dopamine; DLO, dorsolateral orbitofrontal cortex; dHP, dorsal part of hippocampus; DLS, dorsolateral striatum; DMS, dorsomedial striatum; DOPAC, 3,4-dihydroxyphenylacetic acid; DR, dorsal raphe nucleus; dHY, dorsal hypothalamus; EPN, entodepuncular nucleus; Hb, Habenula; HPLC, high-pressure liquid chromatography; HVA, homovanillic acid; IL, infralimbic cortex; LO, lateral orbitofrontal cortex; M2, motor cortex M2; MD, mediodorsal thalamus; MO, medial orbitofrontal cortex; MR, median raphe nucleus; NA, noradrenalin; NAc, nucleus accumbens; pCg, posterior cingulate cortex; pins, posterior insular cortex; PL, prelimbic cortex; 5-HIAA, 5-hydroxyindole-3-acetic acid; 5-HT, 5-hydroxytryptamine; serotonin; 5-HT_2C_R, Serotonin2C receptors; shell, shell of the nucleus accumbens; SN, substantia nigra; STN, subthalamic nucleus; vHP, ventral part of hippocampus; vHY, ventral hypothalamus; VLS, ventrolateral striatum; VMS, ventromedial striatum; VTA, ventral tegmental area.
